# High-pressure high-temperature synthesis of NdRe_2_

**DOI:** 10.3389/fchem.2024.1259032

**Published:** 2024-04-16

**Authors:** Zain Hussein, Nazanin Kazemiasl, Kenan Hussaini, Lia Vaquero, Olga Barkova, Vadym Drozd, Stella Chariton, Vitali Prakapenka, Irina Chuvashova

**Affiliations:** ^1^ Department of Physics, Florida International University, Miami, FL, United States; ^2^ Department of Chemistry and Biochemistry, Florida International University, Miami, FL, United States; ^3^ Department of Mechanical and Materials Engineering, Florida International University, Miami, FL, United States; ^4^ Applied Research Center, Florida International University, Miami, FL, United States; ^5^ Center for Advanced Radiation Sources, The University of Chicago, Chicago, IL, United States

**Keywords:** diamond anvil cell, high pressure high temperature, laser heating, neodymium rhenium, crystal structure, synthesis, laves phases, nuclear waste

## Abstract

In this study, we report the synthesis of a new cubic neodymium-rhenium metallic alloy NdRe_2_ through the utilization of high pressure and laser heating in a diamond anvil cell. NdRe_2_ crystallizes in the $Fd\bar{3}m$ space group with a lattice parameter equal to 7.486 (2) Å and Z = 8 at 24 (1) GPa and 2,200 (100) K. It was studied using high-pressure single-crystal X-ray diffraction. The compound crystallizes in the cubic MgCu_2_ structure type. Its successful synthesis further proves that high-pressure high-temperature conditions can be used to obtain alloys holding a Laves phase structure. *Ab initio* calculations were done to predict the mechanical properties of the material. We also discuss the usage of extreme conditions to synthesize and study materials present in the nuclear waste.

## 1 Introduction

The management of nuclear waste is a complex and multifaceted issue that requires meticulous attention to safety for long-term sustainability. Nuclear energy produces sizeable quantities of nuclear waste (World Nuclear Association, 2021; U.S. Energy Information Administration, 2022), which can impose danger if not properly contained. In this context, materials science plays a vital role in developing innovative solutions for the safe storage and study of nuclear waste (Beierschmitt et al., 2017).

Rare-earth (RE) compounds, a class of materials with unique properties, play a pivotal role in forming substances to solve the nuclear waste challenges the country faces (Chong et al., 2022). Depending on the level of waste, the primary types of materials used include glass, ceramics, cement, and various composites (Hyatt and Ojovan, 2019; Rafiuddin et al., 2022). Stored nuclear waste holds varying levels of lanthanides and actinides that can form unknown compounds depending on their environment. As actinides exhibit high radioactivity, studying their compounds can pose safety risks that prevent a thorough analysis. Lanthanides offer a safer alternative. As both are in the *f*-block, actinides can be modeled by their lanthanide counterpart to safely study potential formations occurring in stored waste.

Among the 4*f* elements, or the lanthanides, neodymium has been of particular interest due to its remarkable magnetic properties (Behrendt et al., 1957), ductility (Wu et al., 2013), and its influence as a dopant in various compounds (Roy et al., 2012; Butenkov et al., 2023). More notably, neodymium is commonly known for its involvement in the permanent magnet Nd_2_Fe_14_B (Boller and Oesterreicher, 1984) and in the lasing medium of an Nd:YAG laser. Alongside its popular uses, neodymium can be used to substitute the actinide uranium. Since the two hold the same number of electrons within the outermost shell, they may exhibit similar bonding formations in their respective compounds. In addition, rhenium can be used to study compounds formed between technetium and actinides during containment. Technetium, a long-lived radioactive element produced during nuclear fission, is specifically targeted for immobilization due to its radioactive nature and potential environmental impact (Hartmann et al., 2011). Technetium shares similar chemistry to rhenium (Darab and Smith, 1996), allowing rhenium to model technetium during experiments. As uranium and technetium are radioactive, direct studies prove to be challenging due to the inherent safety risks. Using similar nonradioactive elements allows us to mimic the radioactive compounds found in nuclear waste for further study.

Laves phase compounds have previously been of immense interest due to their potential applications, such as usage in superalloys and high-temperature steels (Stein and Leineweber, 2021). A large portion of known Laves phase compounds hold an RE element (Villars and Calvert, 1991), and while many are known, there may be an abundance of unknown compounds. Within the neodymium rhenium system, only the hexagonal NdRe_2_ (space group *P*6_3_ */mmc*) (Elliott, 1964) has been previously synthesized. This hexagonal structure is common among the lanthanides (excluding La, Ce, Pm) (Elliott, 1964; Savitskii and Khamidov, 1965) and some actinides (Th, U, Np, Pu) (Giorgi and Szklarz, 1970; Lam and Mitchell, 1972; Haines et al., 1976). It is also seen in several RE-Tc (Darby et al., 1962; Darby et al., 1964; Szklarz and Giorgi, 1981) and actinide-Tc (Darby et al., 1965) compounds. RE-Re alloys have been previously synthesized through arc-melting and liquid-phase sintering (Elliott, 1964). While useful, these methods often have difficulties in providing pure crystalline phases. High-pressure high-temperature (HPHT) synthesis could be a great alternative. Compared to other methods, HPHT synthesis allows for greater control of sample composition and enables us to obtain metastable materials. In addition, its ease of use and compatibility with X-ray diffraction and spectroscopy tools encourage extensive exploration of material behavior under extreme conditions.

Multiple high-pressure studies were conducted on various compounds holding RE and transition metal elements. This includes the HPHT synthesis of YbZn_2_ (Salamatin et al., 2023) and REFe_2_ (RE = Pr, Nd, Yb) (Cannon et al., 1972), the pressure-induced structural transitions in R_1-x_Ni_2_ (R = Y, Sm, Gd, Tb) (Gratz et al., 1996), and the pressure-induced disordering of vacancies in RNi_2_ (R = Y, Sm, Gd, Tb) (Lindbaum et al., 2002). While useful, detailed studies on the HPHT synthesis of RE rhenium alloys have been scarce; therefore, it is essential to determine how the system behaves and develops at elevated conditions.

In this work, we used the advantages provided by single-crystal high-pressure crystallography (Bykova, 2014; Bykova et al., 2015) to synthesize NdRe_2_ for the first time and determine its crystal structure at 24 (1) GPa and 2,200 (100) K. *In situ* X-ray diffraction (XRD) was used in a laser-heated diamond anvil cell (DAC) to characterize the synthesized single-crystal compound.

## 2 Materials and methods

### 2.1 Diamond anvil cell experiment

A mini BX80-type DAC (DACTools, 2012) with diamonds with culet diameters of 100 µm was used in high-pressure experiments. Rhenium gaskets were squeezed between the diamonds to create an indentation with a thickness of 19 µm. A round hole of 52 µm in diameter was then drilled in the center of the indentation. A preselected single crystal of γ-B of size 8 µm and a piece of Nd (commercially purchased from Fischer Scientific, 99.9% purity) of size 10 µm were placed next to each other in the center of the hole. Neon served as the pressure transmitting medium and was loaded at 13 IDD at the Advanced Photon Source (APS) at Argonne National Lab (ANL) (Rivers et al., 2008; Sutton et al., 2022). Alongside the sample, a 3 µm Au piece and a 10 µm ruby ball (DACTools, LLC) were placed near the sample and used as pressure markers. Using ruby fluorescence (Dewaele et al., 2008) and the XRD of Au and He (Fei et al., 2007), pressure values were determined.

### 2.2 Single-crystal X-Ray diffraction

*In situ* XRD measurements were performed at the 13ID-D beamline of GSECARS at APS, ANL using a wavelength λ = 0.2952 Å (42 keV), and the beam size was 3.8 × 2.3 µm^2^. The diffraction data were collected using the Dectris Pilatus3 X CdTe 1M pixel array detector. During the measurement, 140 frames were collected in the scanning range of ± 35° with a step of 0.5° and the exposure time set to 1 s per step. Using the double-sided laser-heating setup at the 13ID-D beamline, the sample was heated to 2,200 (100) K. The laser was set to burst mode with a heating duration of one or 3 s. XRD measurements were taken before, during, and after laser heating. After each laser heating period, the sample was mapped around the heated spot to identify if the reaction occurred. Then single-crystal data were collected in a few spots for further analysis of the phase composition. Initially, pressure in the DAC was increased to 8.0 (5) GPa, but XRD data showed that the sample did not crystallize sufficiently and was not optimal for single-crystal analysis. To overcome this issue, the pressure was further increased to 24 (1) GPa, and after laser heating, we observed a notable reaction.

CrysAlisPro (Rigaku Oxford Diffraction, 2019) was used to perform data reduction and determine the lattice parameters, which can be found in Table 1. Structure solution and refinement were done using JANA 2006 (Petříček et al., 2014) implemented with SHELXT (Sheldrick, 2015b) and SHELXL (Sheldrick, 2015a). DAFi (Aslandukov et al., 2022) was used to search for and identify single-crystal domains in the XRD dataset. Crystal structure visualization was made using VESTA (Momma and Izumi, 2011) and Diamond software (Putz and Brandenburg, 1999).

**TABLE 1:** Crystallographic data for NdRe_2_.

Empirical formula	NdRe_2_
Crystal system	Cubic
Space group	$Fd\bar{3}m$
Space group number	227
a (Å)	7.486 (2)
V (Å^3^)	419.5 (2)
Z	8
Density ( $\frac{g}{{cm}^{3}}$ )	16.3602
R_int_	0.046
R_1_	0.034
GOF(obs), GOF(all)	4.55, 4.51
No. of total reflections	62
No. of parameters	4

### 2.3 Scanning electron microscopy

**FIGURE 1:**
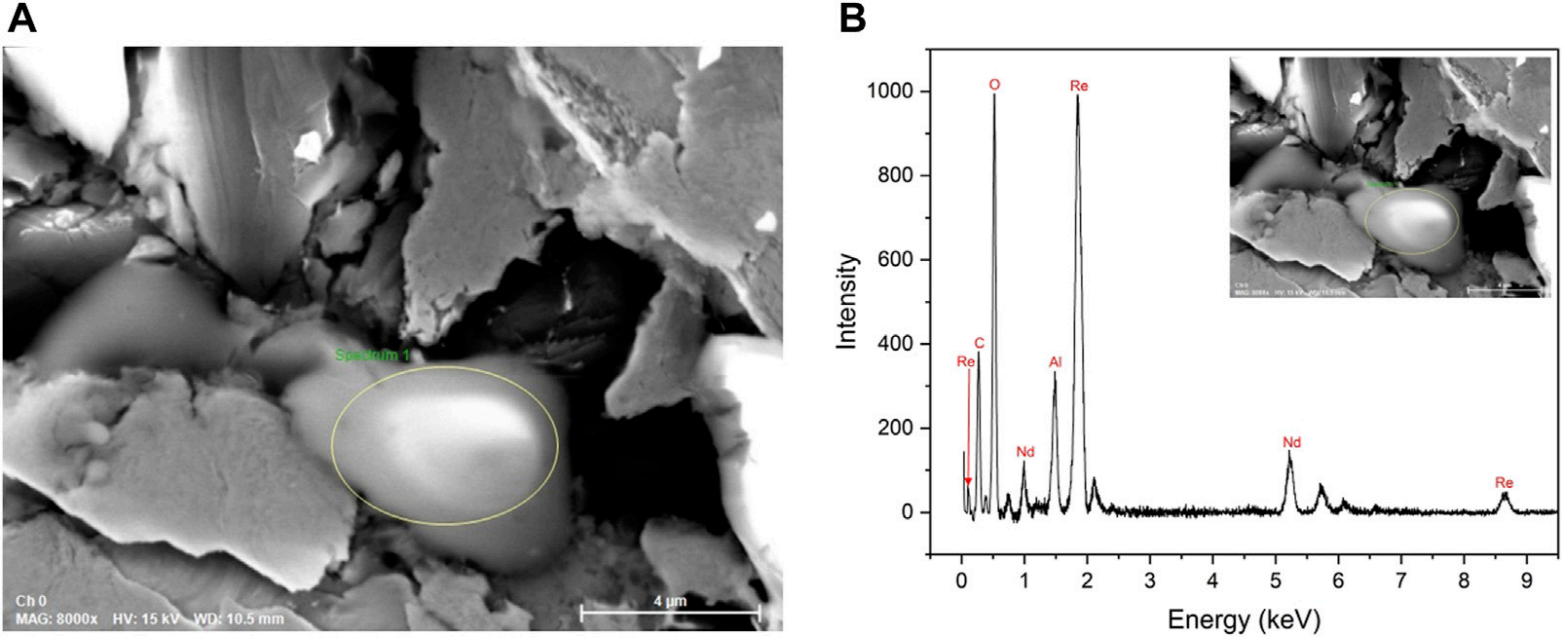
**(A)** SEM image of the sample with the circled area of the sample; **(B)** EDS spectrum of the circled site. We can see Nd, Al_2_O_3_ (ruby), and Re (gasket). Carbon tape was used as a mount. Boron is not detectable via EDS.

The sample was decompressed to ambient pressure and recovered after the experiment. The composition of the sample was analyzed by means of scanning electron microscopy (SEM) using the JOEL JSM-IT500HR scanning electron microscope (JEOL USA, Inc., Peabody, MA, USA). The chemical composition was checked at 15 kV using energy-dispersive X-ray spectroscopy (EDS) of QUANTAX EDS System with XFlash 6,160 detector (Bruker Nano GmbH, Berlin, Germany). The SEM image is shown in Figure 1A, and the resulting spectrum is shown in Figure 1B.

### 2.4 Ab initio calculations

Density functional theory (DFT) calculations were performed using Vienna *ab initio* simulation package (VASP 6.3) (Kresse and Furthmüller, 1996a; Kresse and Furthmüller, 1996b). Generalized Gradient Approximation (GGA) was used to describe exchange and correlation effects. The Perdew-Burke-Ernzerhof functional (Perdew et al., 1996) for the exchange-correlation term was used. Electron wave functions were expanded by plane wave with a cutoff energy of 700 eV. Monkhorst and Pack (1976) k-point grids were set as 11 × 11 × 11. Atomic relaxation was performed until the change in the electronic and ionic steps were less than 10^–7^ eV and 10^–6^ eV, respectively. VASPKIT package (Wang et al., 2021) was used to extract and analyze the VASP raw output files. Elastic constants were determined using energy-strain method.

## 3 Results and discussion

**FIGURE 2:**
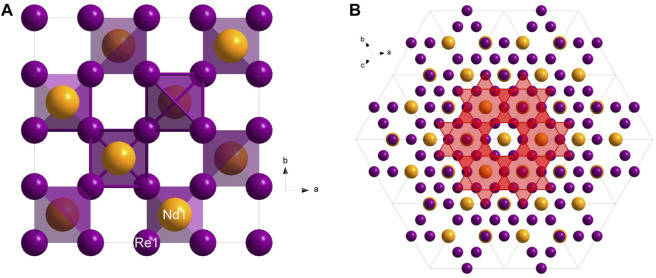
**(A)** View along the c*-plane of the NdRe_2_; **(B)** View along [111] showing the Kagome net pattern.

The newly synthesized NdRe_2_ crystallized at 24 (1) GPa in the centrosymmetric cubic $Fd\bar{3}m$ space group with the unit cell parameter *a* = 7.486 (2) Å. Its structure consists of neodymium atoms surrounded by a network of rhenium tetrahedra (Figure 2A). Common with Laves phase compounds, a Kagome net can be seen along the [111] direction (Figure 2B). The crystallographic data for the newly synthesized NdRe_2_ can be found in Table 1 and its atomic coordinates are in Table 2.

**TABLE 2:** Atomic positions for the NdRe_2_.

Atom	Wyckoff site	x	y	z
Nd	8a	0.375	0.875	0.375
Re	16d	0.25	0.5	0.25

During the initial single-crystal structure solution and refinement, we used only neodymium and boron, however, our R_1_ value was never lower than 12% and the residual electron density was very high. To solve this problem, we decided to verify the composition of the synthesized compound using the EDS analysis. Figures 1A, B shows the SEM image of the sample and the spectrum of its composition, respectively. Since the EDS analysis was conducted after sample recovery and the sample in the rhenium gasket was attached to the carbon tape, the precise location of the observed reaction was unknown. Consequently, multiple spots on both sides of the sample were analyzed. In addition to neodymium, the EDS spectrum showed the presence of rhenium and aluminum oxide. The sample was placed close to the ruby (Al_2_O_3_) pressure marker and the rhenium gasket, which led to the present compound formation under high pressure and laser heating. Carbon tape was used in the EDS analysis as a mount, which does not allow us to determine if carbon was involved during the synthesis. Although boron was placed in the DAC during the experiment, its presence is not detectable using EDS. Prior to the synthesized compound NdRe_2_, several other compositions were attempted during the structure solution. Despite our best efforts, it was shown that NdRe_2_ was synthesized in the experiment.

Due to the limited opening angle of the DAC and, therefore, incomplete datasets, goodness of fit (GOF) values were larger than expected. While additional atom placements were attempted, the GOF value would only marginally decrease while substantially increasing the R_1_ value. The atom’s thermal parameters were refined in anisotropic approximation and had a data/parameter ratio of 15.5. The residual electron density peaks ranged between 0.5–1.8 e/Å^3^, indicating that no atoms were missing from the structure.

**FIGURE 3:**
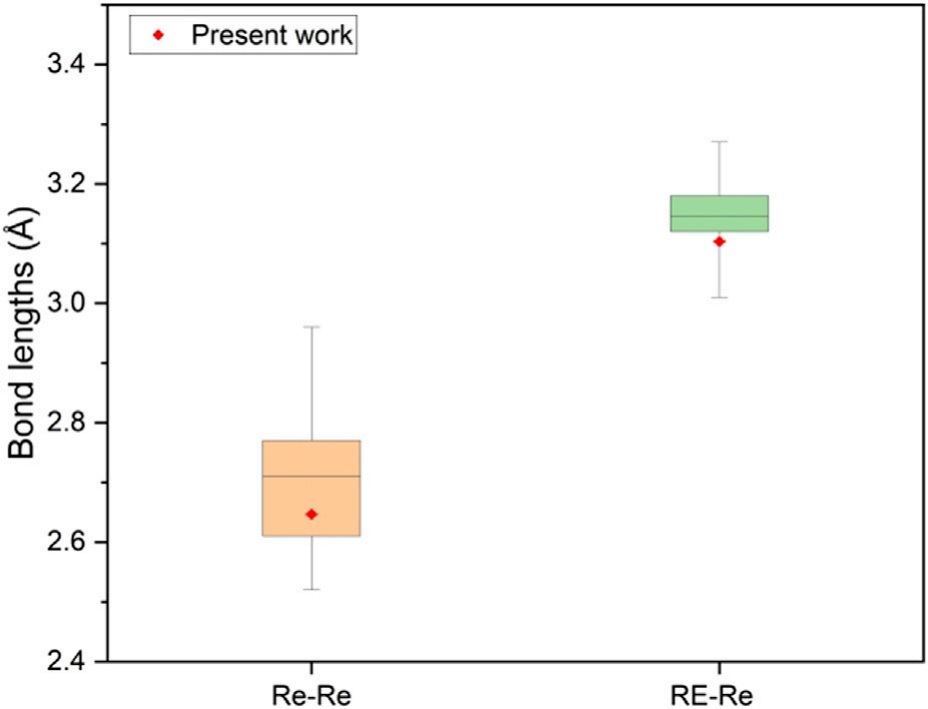
Comparison of interatomic distances of RE-Re and Re-Re in NdRe_2_ with previous studies, which can be found in Supplementary Table S1.

The compound synthesized in this study is the first single crystal obtained at high pressure conditions and one of two RE-Re compounds with a Laves structure to be observed to crystallize in the space group $Fd\bar{3}m$ . The other compound in the same space group is PrRe_2_ (Savitskii and Khamidov, 1965). As the new cubic NdRe_2_ was synthesized using HPHT, we can compare how the interatomic distances vary in our structure *versus* similar compounds synthesized through other methods at ambient pressures (see Figure 3; Supplementary Table S1). In cubic NdRe_2_, we have the interatomic distance between Nd and Re as 3.1035 (14) Å and within the Re tetrahedra, the Re-to-Re distance is 2.6467 (11) Å. In comparison to the cubic PrRe_2_, the cubic NdRe_2_ has shorter Nd-Re and Re-Re bond lengths, which is expected due to HPHT conditions. Relative to all other known RE-Re compounds, the Nd-Re and Re-Re bond lengths are within range.

**FIGURE 4:**
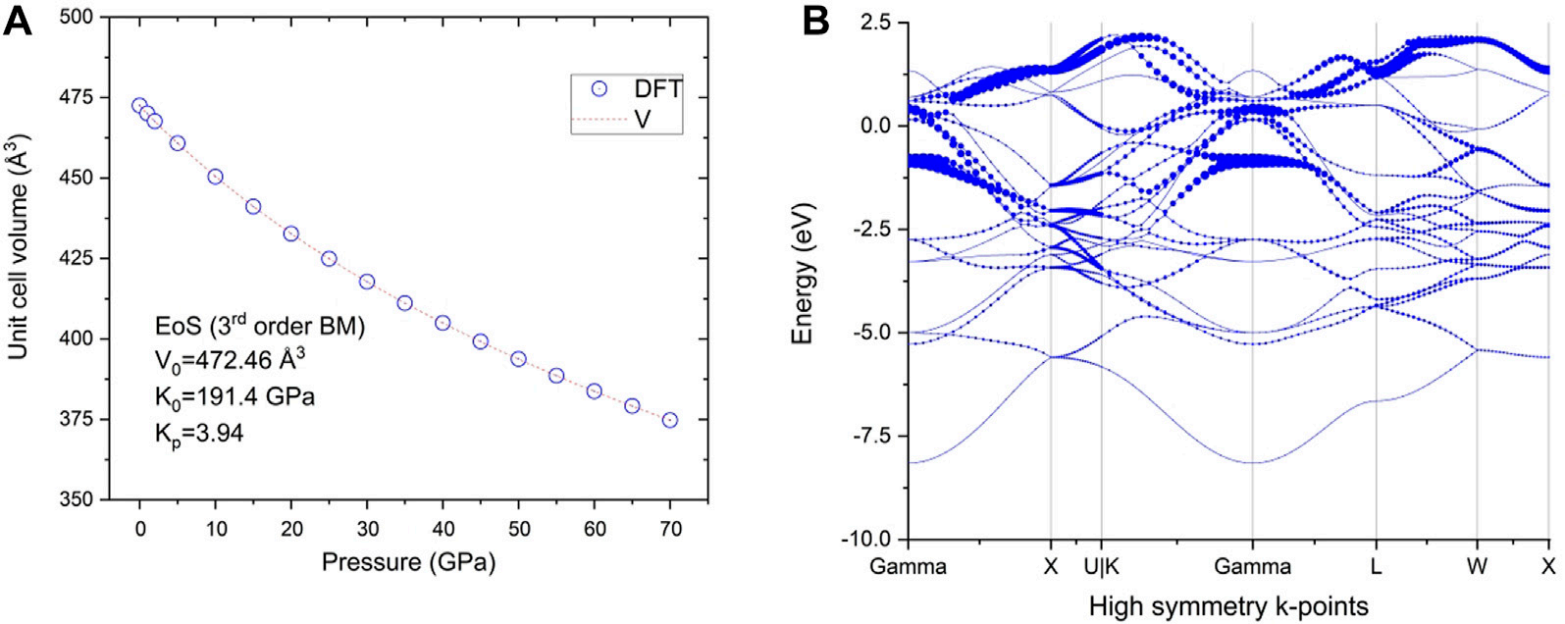
**(A)** Calculated P-V curve for NdRe_2_ (symbols) and the fit to Birch–Murnaghan (third order) equation of state; **(B)** Band structure of NdRe_2_ at ambient pressure.

The calculated mechanical properties of NdRe_2_ can be found in Tables 3, 4. As the structure is cubic, only three independent elastic constants were needed to calculate the compound’s mechanical properties, such as the bulk and shear modulus. With a Young’s and shear moduli of approximately 53 GPa and 18 GPa, respectively, NdRe_2_ is located between the only two calculated moduli in the RE-Re system, PrRe_2_ and YRe_2_ (Jain et al., 2013; de Jong et al., 2015). The equation of state (EOS) (Figure 4A) provides a bulk modulus of 191.4 GPa. While NdRe_2_ has a moderate hardness and low stiffness, its B/G value shows that it has high ductility. This corresponds to the positive value of the Cauchy pressure, which indicates that NdRe_2_ exhibits metallic bonding (Pettifor, 1992; Suetin et al., 2010; Wu et al., 2014). The average wave velocity of 1,275 m/s corresponds to the relatively low Debye temperature of 140.6 K, showing that NdRe_2_ has low thermal conductivity.

**TABLE 3:** Calculated mechanical properties for NdRe_2_. The red headers correspond to the anisotropic mechanical properties of the bulk single-crystal and the blue headers correspond to the average mechanical properties of the bulk polycrystal.

Properties	Min	Max	Anisotropy	Voight	Reuss	Hill
Bulk modulus (GPa)	188.714	188.714	1	188.71	188.714	188.714
Young’s modulus (GPa)	45.840	58.038	1.266	53.19	52.463	52.827
Shear modulus (GPa)	15.705	20.037	1.276	18.30	18.045	18.174
Poisson’s ratio	0.358	0.546	1.523	0.45	0.454	0.453
Linear compressibility (TPa^-1^)	1.766	1.766	1	-	-	-
P-wave modulus (GPa)	-	-	-	213.12	212.775	212.947
Pugh’s ratio (B/G)	-	-	-	10.31	10.458	10.384
Vickers hardness (GPa)	-	-	-	0.51	0.495	0.501

**TABLE 4:** Calculated elastic constants C_ij_ and several mechanical properties of the bulk polycrystal.

C_11_ (GPa)	209.6530
C_22_ (GPa)	178.2450
C_44_ (GPa)	20.0366
Cauchy pressure (GPa)	158.2
Kleinman’s parameter	1.47
Universal elastic anisotropy	0.07
Longitudinal wave velocity (m/s)	3,829.055
Transverse wave velocity (m/s)	1,118.628
Average wave velocity (m/s)	1,275.232
Debye temperature (K)	140.6

In addition to the mechanical properties, the band structure at ambient pressure (Figure 4B) of NdRe_2_ was calculated. As discussed elsewhere (Jovanovic and Schoop, 2022), Kagome lattices and their band structures can differ depending on the atomic bonding present in the crystal structure of a material. The lattice types are divided between a linked and separated lattice, with the latter being subdivided into categories of interacting, semi-interacting, and isolated compounds. The calculated band structure of NdRe_2_ indicates that no Kagome bands are present as it belongs to the cubic MgCu_2_ structure type. Despite this, experimental studies are needed to study the magnetic properties of the material.

## 4 Conclusion

We report the successful synthesis of a neodymium-rhenium alloy with the formula NdRe_2_. The structure was obtained by means of single-crystal X-ray diffraction in a diamond anvil cell at 24 (1) GPa and laser heated up to 2,200 (100) K. Its cubic structure belongs to the $Fd\bar{3}m$ space group. The formation of NdRe_2_ lends itself to being an interesting compound of study for nuclear waste immobilization. As NdRe_2_ can be used to mimic nuclear waste compounds involving technetium and uranium, the potential behavior and properties of these compounds can be safely studied.

## Data Availability

The datasets presented in this study can be found in online repositories. The names of the repository/repositories and accession number(s) can be found below: the joint CCDC/FIZ Karlsruhe online deposition service: https://www.ccdc.cam.ac.uk/structures/? The deposition number CSD-2309202.
