# Supplementary material for: High-pressure high-temperature synthesis of NdRe2
Source: Front Chem. 2024 Apr 16;12:1259032. doi: 10.3389/fchem.2024.1259032 (PMC11058645; doi:10.3389/fchem.2024.1259032)
Supplement: Supplementary file 1 [file Table1.DOCX]

Supplementary Material

High-Pressure High-Temperature Synthesis of NdRe_2_

Zain Hussein^1*^, Nazanin Kazemiasl^2^, Kenan Hussaini^2^, Lia Vaquero^2^, Olga Ibragimova^2^, Vadym Drozd^3,4^, Stella Chariton^5^, Vitali Prakapenka^5^, Irina Chuvashova^1,2*^

^1^Department of Physics, Florida International University, Miami, Florida, United States
^2^Department of Chemistry and Biochemistry, Florida International University, Miami, Florida, United States
^3^Department of Mechanical and Materials Engineering, Florida International University, Miami, Florida, United States

^4^Applied Research Center, Florida International University, Miami, Florida, United States
^5^Center for Advanced Radiation Sources, The University of Chicago, Chicago, Illinois, United States

*** Correspondence:**

Irina Chuvashova

[irina.chuvashova@fiu.edu](mailto:irina.chuvashova@fiu.edu)

Zain Hussein

[zhuss009@fiu.edu](mailto:zhuss009@fiu.edu)

**Table S1.** Interatomic distances (Å) in NdRe_2_ alongside published lanthanide and actinide compounds (RE = lanthanides and actinides).

| **Compounds** | **Space Group** | **RE-Re** | **Re-Re** | **P (GPa)** | **Reference** |
| --- | --- | --- | --- | --- | --- |
| NdRe_2_ | $Fd\overline{3}m$ | 3.1035(14) | 2.6467(11) | 24(1) | Present work |
| ScTc_2_ | $P6_{3}/mmc$ | 3.07-3.08 | 2.53-2.70 | Ambient | (Darby et al., 1962;Szklarz and Giorgi, 1981) |
| ScRe_2_ | $P6_{3}/mmc$ | 3.06-3.10 | 2.56-2.72 | Ambient | (Kripyakevich et al., 1963;Szklarz and Giorgi, 1981) |
| Sc_5_Re_24_ | $I\overline{4}3m$ | 2.78-3.24 | 2.53-2.92 | Ambient | (Kripyakevich et al., 1963) |
| YTc_2_ | $P6_{3}/mmc$ | 3.14-3.16 | 2.60-2.75 | Ambient | (Darby et al., 1964) |
| YRe_2_ | $P6_{3}/mmc$ | 3.15-3.17 | 2.64-2.77 | Ambient | (Compton and Matthias, 1959) |
| PrRe_2_ | $Fd\overline{3}m$ | 3.25 | 2.77 | Ambient | (Savitskii and Khamidov, 1965) |
| PrRe_2_ | $P6_{3}/mmc$ | 3.21-3.25 | 2.72-2.83 | Ambient | (Savitskii and Khamidov, 1965) |
| NdRe_2_ | $P6_{3}/mmc$ | 3.19-3.23 | 2.70-2.81 | Ambient | (Elliott, 1964) |
| SmRe_2_ | $P6_{3}/mmc$ | 3.19-3.20 | 2.67-2.78 | Ambient | (Elliott, 1964) |
| EuRe_2_ | $P6_{3}/mmc$ | 3.16-3.22 | 2.68-2.82 | Ambient | (Elliott, 1964) |
| GdRe_2_ | $P6_{3}/mmc$ | 3.13-3.16 | 2.63-2.76 | Ambient | (Elliott, 1964) |
| TbTc_2_ | $P6_{3}/mmc$ | 3.14-3.16 | 2.60-2.75 | Ambient | (Darby et al., 1964) |
| TbRe_2_ | $P6_{3}/mmc$ | 3.15-3.18 | 2.64-2.77 | Ambient | (Elliott, 1964) |
| DyTc_2_ | $P6_{3}/mmc$ | 3.13-3.15 | 2.59-2.72 | Ambient | (Darby et al., 1964) |
| DyRe_2_ | $P6_{3}/mmc$ | 3.14-3.17 | 2.64-2.77 | Ambient | (Elliott, 1964) |
| HoTc_2_ | $P6_{3}/mmc$ | 3.13-3.14 | 2.59-2.74 | Ambient | (Darby et al., 1964) |
| HoRe_2_ | $P6_{3}/mmc$ | 3.13-3.16 | 2.63-2.77 | Ambient | (Elliott, 1964) |
| ErTc_2_ | $P6_{3}/mmc$ | 3.12-3.13 | 2.58-2.74 | Ambient | (Darby et al., 1964) |
| ErRe_2_ | $P6_{3}/mmc$ | 3.13-3.16 | 2.62-2.76 | Ambient | (Elliott, 1964) |
| TmTc_2_ | $P6_{3}/mmc$ | 3.11-3.12 | 2.57-2.74 | Ambient | (Darby et al., 1964) |
| TmRe_2_ | $P6_{3}/mmc$ | 3.13-3.17 | 2.62-2.79 | Ambient | (Elliott, 1964) |
| YbRe_2_ | $P6_{3}/mmc$ | 3.12-3.13 | 2.61-2.68 | Ambient | (Elliott, 1964) |
| LuTc_2_ | $P6_{3}/mmc$ | 3.10-3.11 | 2.56-2.73 | Ambient | (Darby et al., 1964;Szklarz and Giorgi, 1981) |
| LuRe_2_ | $P6_{3}/mmc$ | 3.12-3.16 | 2.61-2.78 | Ambient | (Elliott, 1964) |
| ThTc_2_ | $P6_{3}/mmc$ | 3.08-3.42 | 2.57-2.89 | Ambient | (Darby et al., 1965) |
| ThRe_2_ | $P6_{3}/mmc$ | 3.15-3.31 | 2.65-2.84 | Ambient | (Dwight, 1961) |
| URe_2_ | $P6_{3}/mmc$ | 3.07-3.27 | 2.64-2.84 | Ambient | (Lam and Mitchell, 1972) |
| URe_2_ | Cmcm | 2.93-3.32 | 2.53-2.96 | Ambient | (Haines et al., 1976) |
| NpRe_2_ | $P6_{3}/mmc$ | 3.01-3.20 | 2.52-2.80 | Ambient | (Lam and Mitchell, 1972) |
| PuRe_2_ | $P6_{3}/mmc$ | 3.02-3.35 | 2.67-2.91 | Ambient | (Haines et al., 1976) |

**References**

Compton, V.B., and Matthias, B.T. (1959). Laves phase compounds of rare earths and hafnium with noble metals. *Acta Crystallographica* 12**,** 651-654, <https://doi.org/10.1107/S0365110X59001918>.

Darby, J.B., Berndt, A.F., and Downey, J.W. (1965). Some intermediate phases in the thorium-technetium and uranium-technetium systems. *Journal of the Less Common Metals* 9**,** 466-468, <https://doi.org/10.1016/0022-5088(65)90132-3>.

Darby, J.B., Lam, D.J., Norton, L.J., and Downey, J.W. (1962). Intermediate phases in binary systems of technetium-99 with several transition elements. *Journal of the Less Common Metals* 4**,** 558-563, <https://doi.org/10.1016/0022-5088(62)90044-9>.

Darby, J.B., Norton, L.J., and Downey, J.W. (1964). Technetium compounds with the MgZn2 structure. *Journal of the Less Common Metals* 6**,** 165-167, <https://doi.org/10.1016/0022-5088(64)90120-1>.

Dwight, A. (1961). Factors controlling the occurrence of Laves phases and AB5 compounds among transition elements. *Trans. ASM* 53**,** 479-500,

Elliott, R.P. (1964). *Laves phases of the rare earths with transition elements.* United States,

Haines, H.R., Mardon, P.G., and Potter, P.E. (1976). *Some constitutional studies on uranium-carbon- and plutonium-carbon-rhenium and technetium systems.* Netherlands: North-Holland,

Kripyakevich, P.I., Kuzma, Y.B., and Protasov, V.S. (1963). CRYSTAL STRUCTURE OF COMPOUNDS IN THE Sc-Re SYSTEM. Vol: No. 4, Journal Name: Dopovidi Akad. Nauk Ukr. RSR; Journal Volume: Vol: No. 4; Other Information: Orig. Receipt Date: 31-DEC-63.

Lam, D.J., and Mitchell, A.W. (1972). Laves phases of actinide elements. *Journal of Nuclear Materials* 44**,** 279-284, <https://doi.org/10.1016/0022-3115(72)90038-4>.

Savitskii, E., and Khamidov, O. (1965). Crystal structure of compounds of rhenium with praseodymium. *Izv. Akad. Nauk SSSR, Neorg. Mater. (Russian)* 1**,** 1621-1622,

Szklarz, E.G., and Giorgi, A.L. (1981). Superconductivity of the Laves phases YTc2, ScTc2 and LuTc2. *Journal of the Less Common Metals* 81**,** 349-351, <https://doi.org/10.1016/0022-5088(81)90041-2>.
