# Supplementary material for: High-pressure high-temperature synthesis of NdRe2
Source: Front Chem. 2024 Apr 16;12:1259032. doi: 10.3389/fchem.2024.1259032 (PMC11058645; doi:10.3389/fchem.2024.1259032)

checkCIF/PLATON report

Structure factors have been supplied for datablock(s) NdRe2_24GPa

THIS REPORT IS FOR GUIDANCE ONLY. IF USED AS PART OF A REVIEW PROCEDURE FOR PUBLICATION, IT SHOULD NOT REPLACE THE EXPERTISE OF AN EXPERIENCED CRYSTALLOGRAPHIC REFEREE.

No syntax errors found.   CIF dictionary   Interpreting this report

Datablock: NdRe2_24GPa

Bond precision: Nd-Nd = 0.0009 A   Wavelength=0.29521

Cell:	a=7.486(2)	b=7.486(2)	c=7.486(2)
	alpha=90	beta=90	gamma=90
Temperature:	298 K		

	Calculated	Reported
Volume	419.5(3)	419.5(2)
Space group	F d -3 m	F d -3 m
Hall group	-F 4vw 2vw	-F 4abvw;2a
Moiety formula	Nd Re2	Nd Re2
Sum formula	Nd Re2	Nd Re2
Mr	516.66	516.70
Dx,g cm-3	16.361	16.360
Z	8	8
Mu (mm-1)	13.723	13.808
F000	1680.0	1680.0
F000’	1642.84	
h,k,lmax	12,12,12	12,12,9
Nref	74	62
Tmin,Tmax	0.933,0.933	0.586,1.000
Tmin’	0.933	

Correction method= # Reported T Limits: Tmin=0.586 Tmax=1.000
AbsCorr = MULTI-SCAN

Data completeness= 0.838   Theta(max)= 14.940

R(reflections)= 0.0337(61)   wR2(reflections)= wR= 0.0468(62)

S = 4.510   Npar= 4

```
The following ALERTS were generated. Each ALERT has the format
       test-name_ALERT_alert-type_alert-level.
Click on the hyperlinks for more details of the test.
```

Alert level B

```
GOODF01_ALERT_2_B  The least squares goodness of fit parameter lies
            outside the range 0.60 <> 4.00
            Goodness of fit given =      4.510
```

Alert level C

```
PLAT029_ALERT_3_C _diffrn_measured_fraction_theta_full value Low .      0.970 Why?
PLAT127_ALERT_1_C Implicit Hall Symbol  Inconsistent with Explicit -F 4abvw;2 Check
```

Alert level G

```
ABSMU01_ALERT_1_G  Calculation of _exptl_absorpt_correction_mu
                not performed for this radiation type.
PLAT004_ALERT_5_G Polymeric Structure Found with Maximum Dimension          3 Info
PLAT005_ALERT_5_G No Embedded Refinement Details Found  in the CIF     Please Do !
PLAT092_ALERT_4_G Check: Wavelength Given is not Cu,Ga,Mo,Ag,In Ka    0.29521 Ang.
PLAT808_ALERT_5_G No Parseable SHELXL Style Weighting Scheme Found     Please Check
PLAT883_ALERT_1_G No Info/Value for _atom_sites_solution_primary .     Please Do !
PLAT952_ALERT_5_G Calculated (ThMax) and CIF-Reported Lmax Differ.          3 Units
PLAT966_ALERT_5_G Note: Non-Standard (i.e. 2.0) OMIT Threshold  of        3.0 Sig(I)
PLAT984_ALERT_1_G The Nd-f'=   -2.6918 Deviates from the B&C-Value    -2.7056 Check
PLAT984_ALERT_1_G The Re-f'=   -0.9555 Deviates from the B&C-Value    -0.9297 Check
PLAT985_ALERT_1_G The Nd-f"=    0.6393 Deviates from the B&C-Value     0.6359 Check
PLAT985_ALERT_1_G The Re-f"=    1.6560 Deviates from the B&C-Value     1.6424 Check
```

```
   0 ALERT level A = Most likely a serious problem - resolve or explain
   1 ALERT level B = A potentially serious problem, consider carefully
   2 ALERT level C = Check. Ensure it is not caused by an omission or oversight
  12 ALERT level G = General information/check it is not something unexpected

   7 ALERT type 1 CIF construction/syntax error, inconsistent or missing data
   1 ALERT type 2 Indicator that the structure model may be wrong or deficient
   1 ALERT type 3 Indicator that the structure quality may be low
   1 ALERT type 4 Improvement, methodology, query or suggestion
   5 ALERT type 5 Informative message, check
```

checkCIF publication errors

Alert level A

```
PUBL008_ALERT_1_A  _publ_section_title is missing. Title of paper.
PUBL012_ALERT_1_A  _publ_section_abstract is missing.
            Abstract of paper in English.
```

Alert level G

```
PUBL017_ALERT_1_G The _publ_section_references section is missing or
            empty.
```

```
   2 ALERT level A = Data missing that is essential or data in wrong format
   1 ALERT level G = General alerts. Data that may be required is missing
```

Publication of your CIF

You should attempt to resolve as many as possible of the alerts in all categories. Often the minor alerts point to easily fixed oversights, errors and omissions in your CIF or refinement strategy, so attention to these fine details can be worthwhile. In order to resolve some of the more serious problems it may be necessary to carry out additional measurements or structure refinements. However, the nature of your study may justify the reported deviations from journal submission requirements and the more serious of these should be commented upon in the discussion or experimental section of a paper or in the "special_details" fields of the CIF. *checkCIF* was carefully designed to identify outliers and unusual parameters, but every test has its limitations and alerts that are not important in a particular case may appear. Conversely, the absence of alerts does not guarantee there are no aspects of the results needing attention. It is up to the individual to critically assess their own results and, if necessary, seek expert advice.

If level A alerts remain, which you believe to be justified deviations, and you intend to submit this CIF for publication in a journal, you should additionally insert an explanation in your CIF using the Validation Reply Form (VRF) below. This will allow your explanation to be considered as part of the review process.

```
# start Validation Reply Form
_vrf_PUBL008_GLOBAL
;
PROBLEM: _publ_section_title is missing. Title of paper.
RESPONSE: ...
;
_vrf_PUBL012_GLOBAL
;
PROBLEM: _publ_section_abstract is missing.
RESPONSE: ...
;
# end Validation Reply Form
```

If you wish to submit your CIF for publication in Acta Crystallographica Section C or E, you should upload your CIF via the web. If you wish to submit your CIF for publication in IUCrData you should upload your CIF via the web. If your CIF is to form part of a submission to another IUCr journal, you will be asked, either during electronic submission or by the Co-editor handling your paper, to upload your CIF via our web site.

PLATON version of 29/11/2023; check.def file version of 14/09/2023

Datablock NdRe2_24GPa - ellipsoid plot

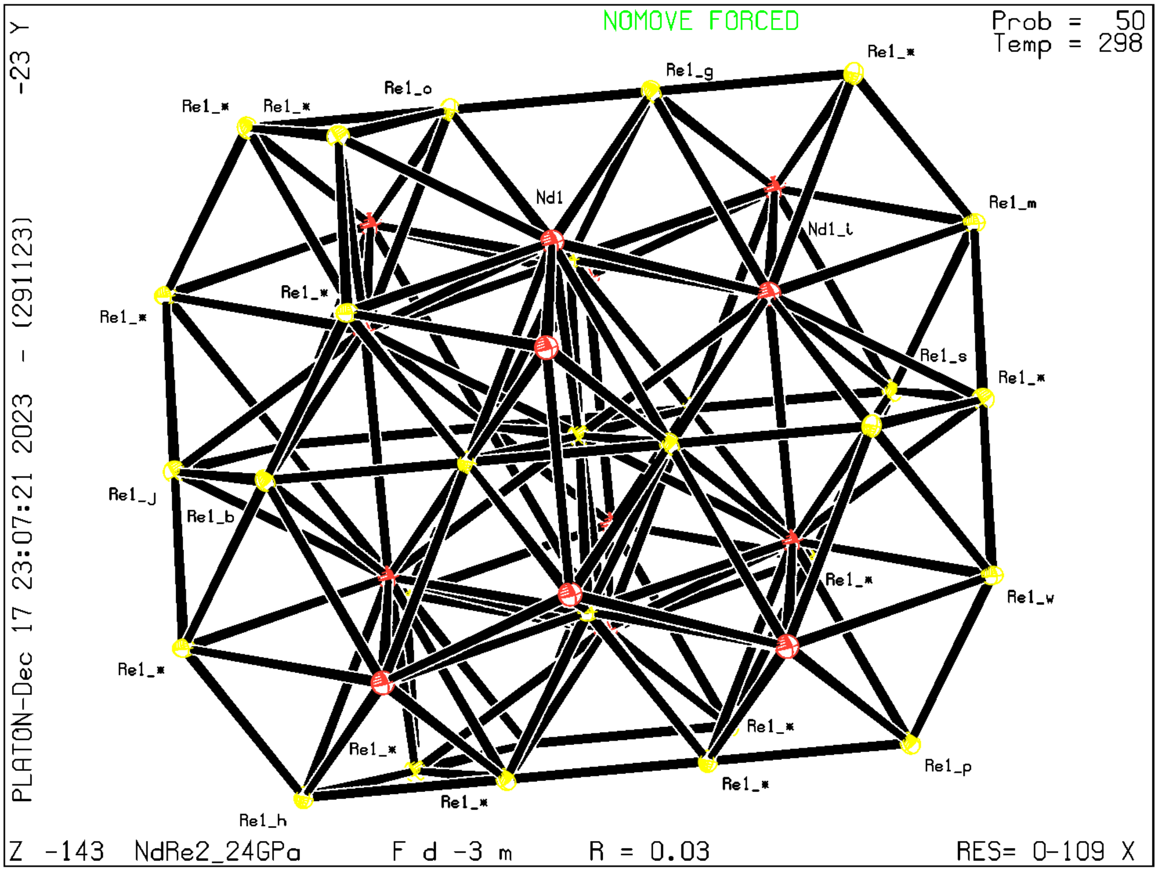

Supplement: Supplementary file 2 [file DataSheet1.pdf]
